# Perinatal Anxiety among Women during the COVID-19 Pandemic—A Cross-Sectional Study

**DOI:** 10.3390/ijerph19052603

**Published:** 2022-02-24

**Authors:** Grażyna Iwanowicz-Palus, Mariola Mróz, Aleksandra Korda, Agnieszka Marcewicz, Agnieszka Palus

**Affiliations:** 1 Chair of Obstetrics Development, Faculty of Health Sciences, Medical University of Lublin, 4-6 Staszica Str., 20-081 Lublin, Poland; spupalus@gmail.com (G.I.-P.); agnieszkamarcewicz@umlub.pl (A.M.); 2 Students’ Scientific Circle at the Chair of Obstetrics Development, Faculty of Health Sciences, Medical University of Lublin, 20-081 Lublin, Poland; kordaaleksandra050@gmail.com; 3 Medical Doctor in Medical Center in NowyDwór Mazowiecki, Faculty of Medicine, Warsaw Medical University, 02-091 Warsaw, Poland; aga1906@gmail.com

**Keywords:** childbirth anxiety, anxiety in pregnancy, COVID-19, SARS-CoV-2, pandemic, support, perinatal care, pregnancy, childbirth school

## Abstract

Introduction: The COVID-19 pandemic has changed the way prenatal education and obstetric care are provided. Pandemic-related anxiety, restrictions, limitations in perinatal care, and the inability to be accompanied by a loved one can have negative psychological consequences for future parents and their child. The aim of this study was to analyze the determinants and assess the anxiety of pregnant women in individual trimesters, as well as to learn about the sources of support and medical personnel proceeding methods. Materials and Methods: This research was conducted as a diagnostic survey, using the State-Trait Anxiety Inventory (STAI), Childbirth Anxiety Questionnaire (CAQ), and a standardized interview questionnaire, on 534 pregnant women in Poland. Resultsand Conclusions: The pregnant women, regardless of the trimester of pregnancy, are characterized by: increased anxiety level influenced by the current epidemiological situation, psychophysical condition, previous maternal experiences, participation in classes preparing for childbirth, organization of perinatal care, their relationship with a partner, and the presence of a loved one during childbirth. A negative correlation was shown between the level of childbirth anxiety and maternal experience, as well as the support of a doctor and midwife.

## 1. Introduction

When faced with situations threatening life or health, negative emotions appear. One of these is anxiety. When it is only moderate, it can increase the motivation to take action. However, as it worsens, it becomes a type of pathological emotion that has a negative impact on the person’s psyche and health [1].

Perinatal anxiety has a significant influence on the health situation of the mother and the development of the child. It is a common problem, as one in ten mothers experience symptoms of anxiety in pregnancy and postpartum [2].

Due to their situation, pregnant women constitute a special group with regard to anxiety over their own health and that of their child. In each of the three trimesters of pregnancy, there are different stress factors thatchange or evolve over the course of the pregnancy. A particular stressor is the approach to childbirth, which is associated with perinatal anxiety. Moreover, a high level of anxiety is also associated with a decrease in the effectiveness of coping strategies [3,4,5].

Stress and anxiety in pregnancy can have a catastrophic effect both on the course of the pregnancy and on the condition of the child. Stress-induced pregnancy complications are a significant cause of morbidity and mortality in mothers and newborns [6,7].

Infants of mothers experiencing perinatal anxiety have a greater risk of developing negative effects. In this group of children, disorders of sleep, interaction with the mother, emotional development, and social relationships are more often observed. The consequences for the mother may be difficulties in breastfeeding and preterm labor [8].

Accurate identification of factors influencing the risk of perinatal anxiety may significantly contribute to the detection of disorders even before pregnancy. Early diagnosis reduces the severity and recurrence of symptoms [9].

Perinatal care in Poland is based on the Standard of Perinatal Care guidelines. This document discusses, inter alia, prenatal education and childbirth procedure. It emphasizes the importance of social, emotional, and informational support for the pregnant woman. It also guarantees the pregnant woman the choice of the place for giving birth, the opportunity of a family midwife, and the benefit of the support of a loved one [10].

The rapid progress of the SARS-CoV-2 pandemic has been a challenge for healthcare systems and has contributed to changes in the manner of delivering prenatal education and maternity care while safeguarding mother and child. Unfortunately, due to the pandemic and prevailing restrictions, access to prenatal education has become difficult. Moreover, there is only limited data on the effects coronavirus has on the pregnant woman and her baby [11,12]. The lack of such important information can also contribute to an increase in uneasiness and perinatal anxiety of pregnant women. In addition, factors such as pandemic-related anxiety, a time of isolation, restrictions, limitations on the process of childbirth and perinatal care, and the impossibility of being accompanied by a loved one have been associated with negative psychological consequences for future parents and their children [13,14].

Available empirical reports have shownthat the COVID-19 pandemic had a significant impact on the mental health of pregnant women. Perinatal anxiety was significantly higher during the pandemic period than before it. Moreover, the previous analyses indicate social support both from relatives and medical staff as a protective factor. The exact determinants of this phenomenon remain a subject of research [15,16,17].

The analysis of determinants of social anxiety in the context of health care system activities-perinatal care and antenatal education was an innovative aspect of our study. An important issue seems to be the assessment of psychophysical condition and its determinants, as well as the determination of correlations between psychological and physical condition and the level of anxiety experienced by pregnant women. Results presented inprevious studies have not determined the relationship between patients’ expectations about labour and care provided by medical staff and perinatal anxiety, so we included this issue in our manuscript.

Due to the importance of the problem of fear of childbirth and the complexity of the topic resulting from the special situation caused by the COVID-19 pandemic, it is very important to assess the determinants of the phenomenon in pregnant women. Obtaining knowledge on the determinants of labour fear in the context of the COVID-19 pandemic might set new directions in psychopreventive actions in the population of pregnant women. Understanding the characteristics and identification of vulnerable groups of women will enable the implementation of appropriate psychoprophylactic interventions. As a result, it might contribute to reducing the risk of sequelae in the form of emotional disorders in the perinatal period.

The main aim of the study was to evaluate perinatal anxiety in pregnant women during the first wave of the COVID-19 pandemic.

Specific objectives:

- Learning about respondents’ opinions on the support received from medical personnel and relatives.
- Assessment of received social support impact on the perinatal anxiety level.
- Assessment of perinatal care and education and its impact on experienced perinatal anxiety level.
- Analysis of the relation between psychological condition and experienced perinatal anxiety level.
- Assessment of the influence of selected obstetric factors on the experienced perinatal anxiety occurrence.

## 2. Materials and Methods

We declare that all procedures performed in studies involving human participants were in accordance with the ethical standards of the institutional and/or national research committee (the Bioethics Committee of the Medical University of Lublin: KE-0254/30-2019) and with the 1964 Helsinki declaration and its later amendments or comparable ethical standards. Informed consent was obtained from all individual participants included in the study.

### 2.1. Study Design and Participants

The study was conducted from 1 March 2020 to 2 June 2020, among women in the first (119), second (170), and third (245) trimesters of pregnancy, availing themselves of check-up visits to an OB-GYN and prenatal education conducted by a midwife or a family school in the territory of the Voivodeship of Lublin (eastern Poland).

The sample size was of a non-probalistic character. The study was conducted in selected medical centers providing free medical care for women under health insurance which is available to all pregnant women in Poland, regardless of their income level. From the beginning of a pregnancy, women in Poland are entitled to physician’s care or midwife care (although in practice this is more rarely chosen). From the twenty-first week of the pregnancy, they can take advantage of free visits to a midwife for prenatal education.

Qualification criteria for the study were: agreement to participate in the diagnostic survey, being at least 18 years of age, and diagnosed with a single pregnancy. Persons undergoing psychotherapy or psychiatric treatment were excluded from the study.

In determining the sample size, we took into consideration the number of births from the beginning of 2020 to the moment of our project’s implementation (January–February 2020). The number of births during this period was 3120, so the minimum number of respondents was calculated at 342 (with a maximum error of 5% and a confidence level of 95%). The respondents were informed that participation in the study was voluntary and anonymous and that the results would be used only for scientific purposes. The course and purpose of the study as well as the method of filling in the questionnaire were discussed with the respondents. Each participant received a questionnaire and an informed consent form. In order to preserve participants’ anonymity, the questionnaires and consent forms were deposited into a ballot-type box, which was opened after the end of the study. 

**Figure 1:**
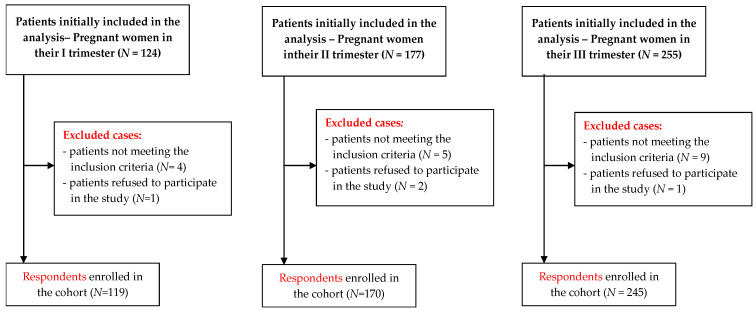
Recruitment process flowchart.

The original goal of the project was to study perinatal anxiety in women in the individual trimesters of pregnancy. Due to the pandemic, which occurred at the same time, the study was adapted to the epidemiological situation and this final version is the one presented in the manuscript. The first complete questionnaires, taking into account aspects of the epidemiological situation, were received on 20 March 2020, and are included in the study.There was a total of 556 participants in the study, out of which four persons did not give consent to participate in the diagnostic survey, plus 18 questionnaires were incomplete or filled in incorrectly. Therefore, 534 questionnaires qualified for statistical analysis. The efficiency ratio of the obtained data was 96.04% (Figure 1).

The study was conducted by diagnostic survey method, with the use of a questionnaire. The research tool was a questionnaire consisting of three sections:

- The State-Trait Anxiety Inventory (STAI) is a tool comprising two scales. The first part of the STAI (x-1) examines the level of anxiety as a current emotional state. It consists of 20 statements, for each of which the respondent chooses one of four possible answers (definitely, probably, probably not, definitely not). The responses to these statements describe the respondent’s feelings while filling out the questionnaire. The second part (x-2) concerns anxiety understood as a personality trait. It also consists of 20 statements that the respondent can answer, using a four-point scale (almost never, sometimes, often, almost always). The responses for this second part provide a picture of how the respondent usually feels [18,19]. The Cronbach’s alpha coefficient for the questionnaire for the studied group was 0.908 (x-1) and 0.869 (x-2), (Supplementary File).
- The Childbirth Anxiety Questionnaire (CAQ): a tool for gaining information on emotions associated with upcoming childbirth. The CAQ is made up of nine statements to which the respondent answers by choosing one of four categories (definitely, probably, probably not, definitely not) to which numerical values are assigned. The higher the score, the greater severity of childbirth anxiety [20]. The Cronbach’s alpha reliability coefficient for the research group was 0.824, (Supplementary File).
- The questionnaire specially prepared for this study takes into consideration the characteristics of the women being researched as well as questions concerning the research topic. The respondents answered on a five-point Likert scale (1—definitely not, 5—definitely yes) on the topics of determinants of childbirth anxiety they felt and healthcare conditions in the time of the SARS-CoV-2 virus pandemic.

### 2.2. Statistical Analysis

SPSS software (IBM SPSS 25 Statistic, Chicago, IL, USA) was used in the data analysis. The analysis of descriptive statistics, chi-square tests of independence, analyses of Pearson’s and rho Spearman’s r correlation, Student’s *t*-tests for independent samples, Mann–Whitney’s tests, one-way ANOVA, and Kruskal–Wallis tests were performed with its help. The level of statistical significance was *p* < 0.05.

## 3. Results

Table 1 presents the characteristics of the women participating in the diagnostic survey, broken down according to the trimester of their pregnancy. Participating in the study were 534 women aged 18 to 48 years old (average age: 27.47 ± 3.92 years), of whom 119 (22.3%) were in their first trimester (1–13 weeks), 170 (31.8%) in the second trimester (14–26 weeks), and 245 (45.9%) in their third trimester (27–40 weeks) of pregnancy (Table 1).

The respondents were dominated by women residing in places within the voivodeship (39.3%), persons with higher education (62.2%), not working during their pregnancy (68.2%), in a married or informal relationship (91.0%), recognizing their material situation as good (73.8%), and not having maternal experience (65.2%). They were also mostly people whose current pregnancy was being attended by an OB-GYN (69.1%), as well as women preparing for childbirth through education, in direct contact with a midwife (27.3%) (Table 1).

In the first stage of the study, the level of anxiety of the respondents was assessed with regard to the stage of pregnancy. The women participating in the study, regardless of pregnancy trimester, were characterized by elevated, high, or very high degrees of anxiety. Most of the women in their second trimester (82.9%) obtained this result, whereas the women in their first (57.2%) or third (58.0%) trimester had very similar anxiety levels. More than half the women stated that their current pregnancy taking place in this epidemiological situation contributes to the anxiety they feel before childbirth (I: 57.2%; II: 56.5%; III: 60.0%). Women in their third semester agreed the most with this opinion (*p* = 0.008). Statistical analysis showed that respondents declaring that their pregnancy concluding in accordance with their previous ideas or plans would not reduce their feelings of anxiety represent a higher level of anxiety (*p* = 0.004), as compared to pregnant women who believe the opposite or who do not have an opinion on this topic (Table 2).

An assessment was made of the attitude of pregnant women regarding support from their loved ones and from medical personnel in the current epidemiological situation. Their responses show that the vast majority of them consider the support of their OB-GYN to be sufficient (I: 76.5%, II: 79.4%, III: 75.5%). Women in their third trimester (42.4%) cited support from the midwife as adequate, while the respondents in the first (52.9%) and second trimester (52.9%) did not have an opinion on this matter. The respondents stated that the support of their loved ones (partner, relatives, friends) is important (I: 95.8%; II: 100%; III: 99.2%, *p* = 0.0260) and, in the current situation, they find it to be adequate (I: 91.6%; II: 90.6%; III: 89.4%). The majority of respondents also gave positive answers to a question about the influence of their relationship with their partner (I: 86.5%; II: 91.8%; III: 93.5%) and the presence of a loved one during childbirth (I: 77.3%; II: 78.2%; III: 78.8%) have on their level of childbirth anxiety (Table 3).

About half of the respondents considered birthing schools or individual prenatal education provided by a midwife to be helpful in preparing physically (I: 61.4%, II: 56.5%, III: 47.3%, *p* = 0.002) and psychologically (I: 71.4%, II: 72.4%, III: 69.3%, *p* < 0.05) for childbirth. According to women in the third trimester of pregnancy (45.7%), childbirth anxiety during the current epidemiological situation was also discussed during the classes, while other respondents did not have an opinion on this subject (*p* = 0.017). When asked whether the topic of coronavirus (I: 77.3%, II: 75.9%, III: 53.5) and childbirth in the current epidemiological situation (I: 78.2%, II: 75.3%, III: 53.1%) were discussed during meetings/videoconferences with a family midwife or in birthing school, most of the respondents answered that they did not have an opinion on this subject because they had not had the opportunity to take advantage of such forms of prenatal education or were not interested in them. Regardless of their stage of pregnancy, the respondents were of the opinion that birthing school and perinatal education are helpful in coping with perinatal anxiety (I: 55.5%, II: 57.0%, III: 57.9%) (Table 3).

The pregnant women taking part in the study were asked to give their opinion on factors thatmight lower childbirth anxiety. Respondents in all three trimesters said that perinatal care (I: 73.1%, II: 78.8%, III: 79.2%) and familiarity with perinatal care standards (I: 58.8%, II: 75.2%, III: 79.2%) had an effect on feelings of anxiety before childbirth (*p* < 0.05). On the other hand, the respondents did not agree with the statement that concluding the pregnancy by Cesarean section would mitigate anxiety (I: 54.6%, II: 51.2%, III: 57.5%) (Table 3).

In the next stage of the study, we looked at the opinions on social support and factors influencing the level of childbirth anxiety. The results were also statistically significant (*p* < 0.05). 

Respondents more highly valuing the support of loved ones, their attending physician, and the midwife providing prenatal education agreed more with the statement that they are in good psychological condition (*p* = 0.001). In turn, the pregnant women saying they considered the support of medical personnel as sufficient assessed their physical condition as good to a greater extent (Table 4).

The statement that their current pregnancy contributes to an increase inanxiety was most strongly agreed to by women who are first-time mothers (M = 3.65) and, toa lesser degree, by respondents whose previous delivery was uneventful (M = 3.17, *p* = 0.005). Furthermore, compared to women in their first pregnancy (M = 2.48) and respondents who had given birth without complications (M = 2.01), pregnant women who had had a delivery burdened with complications (M = 3.12) stated significantly more often that concluding their pregnancy by Cesarean section would reduce feelings of anxiety.

It was also shown that the respondents participating in childbirth preparation classes (M = 24.13) significantly more often (*p* < 0.001) stated that the organization of perinatal care and prenatal education/birthing school reduced childbirth anxiety, compared to those who did not use such activities (M = 21.60), (Table 4).

Table 5 and Table 6 present results of analysis between childbirth anxiety and specific factors: psychophysical condition, maternal experience, manner of conclusion of a previous pregnancy, participation in childbirth preparation classes, and support of medical staff. The data obtained indicate significant relationships among the selected variables (*p* < 0.05).

It has been shown that pregnant women in poor mental (*p* < 0.001) or physical (*p* < 0.001) condition are exposed to a higher level of anxiety as a state and anxiety as a trait than women assessing their psychophysical condition as good (Table 5).

This analysis indicated a statistically significant effect for anxiety as a trait (*p* = 0.019): the women not participating in childbirth preparation classes are characterized by a greater severity of anxiety as a personality trait than are women receiving prenatal education (Table 5).

Analysis of the research showed a statistically significant (*p* < 0.05) negative correlation between the level of childbirth anxiety as a state and anxiety as a trait, as well as the support of an attending OB-GYN(respectively: r = −0.15, r = −0.17) and a family midwife providing prenatal education (r = −0.13, r = −0.18), (Table 6).

The design of the research questionnaire also allowed respondents to freely express themselves about perinatal care, in particular childbirth during the SARS-CoV-2 virus pandemic. Several women in the second or third trimester of pregnancy shared their opinions; a few selected statements are presented below:

“*I am worried about the current epidemiological situation and the impossibility of family members being present for the delivery; even more, I am stressed about giving birth by myself.*”

“*I have brief attacks of hysteria, but they pass quickly.*”

“*The current epidemic greatly increases my anxiety before giving birth. My husband has promised to be with me for the delivery, our due-date is the end of September. Knowing that having family members at the delivery has still not been restored yet at the hospitals in my region causes additional, senseless anxiety and panic. And to what purpose? I am not afraid of a virus, I am afraid of trauma and post-partum depression caused by having my rights, peace, and dreams taken away. I cannot imagine being alone in such a difficult situation as giving birth to my first child.*”

Analysis of the responses shows that for women who had made long efforts to become pregnant and had a difficult gynecological examination, anxiety associated with labor and delivery was concerned more with the health of the child than with their own psychophysical comfort regarding support and help from their loved ones, including being accompanied by their partner:

“*The long years of fighting infertility have certainly influenced my perception of anxiety and childbirth, because I know that I may not have a second chance, so I am more afraid. And now this epidemic…*”

“*More than labor and delivery, I am afraid about successfully carrying the pregnancy, due to an earlier miscarriage and long, in my opinion, attempts to have a baby. My desire for a child is so great that I am not interested in the fact that I will feel pain, I am ready for anything, just to give birth successfully, especially in this situation with coronavirus.*”

## 4. Discussion

In the perinatal period, as a result of the psychological and physiological changes taking place, a woman is particularly exposed to an increased risk of anxiety. The difficult epidemiological situation of the COVID-19 pandemic and the restrictions associated with it, as well as fluctuating socio-economic changes, can additionally increase the spread of psychological problems among perinatal women [21,22].

Women who were pregnant during the first wave of the COVID-19 pandemic, regardless of trimester, were characterized by at least an elevated level of perinatal anxiety.

In our analysis, conducted during thefirst wave of COVID-19 pandemic, the majority of pregnant women were characterized by at least an elevated level of perinatal anxiety.

The respondents claimed that pregnancy in the current epidemiological situation contributes to increased feelings of anxiety.The level of perinatal anxiety experienced by the respondents was also influenced by maternal experience, the course of the previous delivery, and the psychophysical condition.Social support and perinatal care were important for the occurrence of labour anxiety.

In their study, Ahmad et al. observed that during the pandemic the level of anxiety in pregnant women increased in comparison with the period before the epidemic [21].

The findings of this review suggest that the respondents, regardless of which trimester of pregnancy, were characterized by elevated, high, or even very high levels of anxiety. In contrast, Shrestha’s research showed that manifestation of anxiety symptoms was more intense in women in the first trimester of pregnancy [23]. On the other hand, other reports show that the highest level of anxiety was shown by pregnant women in the third trimester of pregnancy [24]. Kahyaoglu, in turn, showed no correlation between the week of pregnancy and the severity of perinatal anxiety [25].

The results obtained by our own research indicate that pregnant women assess the support received by their loved ones as important. A statistically significant relationship was found between the mental condition of pregnant women and the support they received from their loved ones during the COVID-19 pandemic. The effects we present correspond to the reports of Naz et al., whichdemonstrate a strong relationship between family support received by pregnant women and reduction of their feelings of childbirth anxiety. Women who received support from their loved ones declared milder feelings of childbirth anxiety. In turn, respondents who did not receive this kind of support felt a significantly higher level of anxiety [26].

The results of our analysis indicate a relationship between the support received by pregnant women from medical staff and anxiety of the pregnant women. The available research confirms that pregnant women have a particular need for support from medical personnel. This is very relevant during the COVID-19 pandemic. The support provided them reduces stress and anxiety, increasing their quality-of-life assessment. It has a positive effect on psychophysical well-being, reducing the anxiety associated with hospitalization [27,28,29]. According to reports by other authors, the support of medical staff was not able to compensate for the lack of a loved one. This absence caused a feeling of helplessness and intensified perinatal anxiety [30]. The social distancing in force everywhere can constitute a serious problem resulting in psychological discomfort, as social support is of particular importance in buffering the negative effects of stress and anxiety [31].

Our findings indicate that pregnant women attending birthing schools showed less severe anxiety as a personality trait than women not choosing to participate in prenatal education. The outcomes of analyses by Aksoy et al. concur with these results [12]. Other researchers have observed particularly helpful effects from participating in birthing schools among first-time mothers. This group feels great stress in adapting to the role of motherhood. Prenatal education makes possible the preparation of young mothers for a new situation. Moreover, positive effects can be seen in the collaboration of the first-time mother with the obstetric team during childbirth. Thanks to the emotional support of other women in the same situation, a significant reduction of perinatal anxiety comes from the exchange of experiences in organized group activities [32].

Karlström et al. explain the limited effects of participation in childbirth classes among multiparous women by the stronger influence of previous obstetric experiences, which have formed the pregnant woman’s attitude regarding the next birth [33].

Results of research by Swift et al. demonstrated that women who had expressed feelings of childbirth anxiety declared a decrease in them under the influence of participation in birthing school classes [34]. Kuciel et al., conducting research during the COVID-19 pandemic, showed that the knowledge acquired by respondents in prenatal education did not affect their level of perinatal anxiety [35]. Hassanzadeh, guided by the positive effect of studies on the benefits of participation in prenatal classes, suggests implementing participation in birthing training as part of standard prenatal care [36].

Research studies have sought evidence of the influence of the ordering of pregnancies on the mother’s mental health and related factors. Farewell et al. indicated moderate or severe intensification of perinatal anxiety symptoms in more than half of the respondents who were in their first pregnancy [37]. In the group we studied, women in their first pregnancies agreed to the greatest degree with the statement that their current pregnancy was contributing to increased anxiety. This similarity of the results may be related to the natural tendency to fear the unknown, or to an intensified conviction that childbirth is associated with medical intervention.

Our probe showed that pregnant women are not of the opinion that having a Cesarean section eases perinatal anxiety. An analysis conducted by Mehdizadehkashi was dominated by respondents characterized by a high level of anxiety, of whom as many as 39% asked for an elective Cesarean section. In this same group, 86.4% of respondents felt frustrated because of the COVID-19 pandemic [38]. In the reports byMortazavi et al., the main predictor of pregnancy being concluded by Cesarean section was the pregnant woman’s fear of childbirth and the pain associated with it [39]. Research conducted by Størksen also shows that the main reason for a woman choosing Cesarean section without clear medical indication is fear of childbirth [40]. Despite increasingly frequent study results which indicate a growing number of patients awaiting elective Cesarean section, Malhotra demonstrates in his analyses that during the first year of the COVID-19 pandemic, the number of Cesarean sections in New York remained at a level similar to that recorded in the preceding years [41].

The existing research analysis shows how important it is to properly adjust the perinatal care system to the current epidemiological situation. It shows the importance of proper care implementation, despite the difficulties related to, inter alia, restrictions, as well as support from medical staff. Bearing in mind the potential negative psychological consequences of social isolation during the COVID-19 pandemic, there is a need to conduct further research on the determinants of perinatal anxiety and to identify protective factors, the knowledge of which will enable the provision of appropriate care to pregnant women.

### Strengths and Limitations of the Study

The presented results come from an analysis based on a subjective assessment of Level of COVID-19 Anxiety in pregnant women. Although we used scales that are considered sensitive research tools, they are based on subjective feelings and do not include objective criteria of clinical symptoms.It is worth conducting a study where the same analysis for pandemic and non-pandemic situations could be performed to better understand which factor has a greater influence on the level of anxiety (pregnancy, preparation, social support or the pandemic situation itself). Moreover, the study did not include the assessment of individual and sociodemographic characteristics (e.g., low-risk pregnancies, high-risk pregnancies, education, place of residence, self-reported financial standing). This is a cross-sectional study, so no claims can be made about causality.

The advantage of our work is the size of the study group (534 people), and the fact that our questionnaire was delivered to each respondent in person. It should also be emphasized that the study utilized a standardized tool, which allows other authors studying the issue to compare research results and explore the subject.

Despite certain limitations, our study can constitute a reference point for further exploration of the problem of COVID-19-related childbirth anxiety. Moreover, it can make possible a rapid initiation of appropriate psychoprophylactic interventions in a given epidemiological situation.

## Figures and Tables

**Table 1:** Participants’ baseline characteristics.

Participants’ Characteristics		I Trimester	II Trimester	III Trimester	Total
		% (n)	% (n)	% (n)	
Age	<20	2.5 (3)	0.6 (1)	2.4 (6)	1.9 (10)
	20–29	78.2 (93)	64.1 (109)	61.2 (150)	65.9 (352)
	30–39	19.3 (23)	33.5 (57)	34.3 (84)	30.7 (164)
	≥40	-	1.8 (3)	2.0 (5)	1.5 (8)
Residence	urban—province capital	36.1 (43)	38.8 (66)	41.2 (101)	39.3 (210)
	other cities	40.3 (48)	37.6 (64)	33.5 (82)	36.3 (194)
	rural	23.5 (28)	23.5 (40)	25.3 (62)	24.3 (130)
Education	university	63.9 (76)	64.2 (109)	60.0 (147)	62.2 (332)
	other educational stages	36.1 (43)	35.8 (61)	40.0 (98)	37.8 (202)
Professional activity	currently does not work	18.5 (22)	65.9 (112)	81.2 (199)	68.2 (364)
	does not work professionally at all	44.5 (53)	13.5 (23)	6.1 (15)	11.2 (60)
	(she) works	37.0 (44)	20.6 (35)	12.7 (31)	20.8 (110)
Relationship status	married/informal relationship	89.1 (106)	90.0 (153)	92.7 (227)	91.0 (486)
	single	10.9 (13)	10.0 (17)	7.3 (18)	9.0 (48)
Self-reported financial standing	good	73.9 (88)	74.7 (127)	73.1 (179)	73.8 (394)
	bad	26.1 (31)	25.3 (43)	26.9 (66)	26.2 (140)
Having children	no, it’s the first pregnancy	58.8 (70)	63.5 (108)	69.4 (170)	65.2 (348)
	one child	26.9 (32)	28.8 (49)	23.7 (58)	26.0 (139)
	two or more children	14.3 (17)	7.7 (13)	6.9 (17)	8.8 (47)
The person providing care	doctor	67.2 (80)	73.5 (125)	66.9 (164)	69.1 (369)
	midwife	5.9 (7)	1.2 (2)	0.4 (1)	1.9 (10)
	doctor and midwife	25.2 (30)	24.1 (41)	31.8 (78)	27.9 (149)
	she was not under the care of a doctor/midwife	1.7 (2)	1.2 (2)	0.8 (2)	1.1 (6)
Participation in Childbirth Classes	yes—face-to-face meeting with the midwife	30.3 (36)	18.8 (32)	31.8 (78)	27.3 (146)
	yes—video- and teleconferences	0.8 (1)	10.0 (17)	12.3 (30)	9.0 (48)
	no, she did not have the opportunity/possibility	5.9 (7)	14.1 (24)	25.7 (63)	17.6 (94)
	no, she was not interested	12.6 (15)	23.5 (40)	24.1 (59)	21.3 (114)
	has not participated yet but would like to	50.4 (60)	33.5 (57)	6.1 (15)	24.7 (132)

(*n*)—number, %—percentage.

**Table 2:** Anxiety levels of the pregnant women according to pregnancy trimester.

**Pregnancy Trimester**	**Childbirth Anxiety**							
	**Anxiety as a State**				**Anxiety as a Trait**			
	**M**		**SD**		**M**		**SD**	
I trimester	40.97		8.46		40.97		8.77	
II trimester	41.12		9.24		42.35		8.63	
III trimester	42.40		8.42		42.20		8.75	
Statistic	F *=* 1.58 *p* = 0.207 η^2^ = 0.01				F *=* 1.03 *p* = 0.35 η^2^ = <0.01			
**Anxiety Level**	**Pregnancy Trimester**							
	**I**			**II**		**III**		
	**% (n)**			**% (n)**		**% (n)**		
Low	42.8 (51)			37 (63)		42 (103)		
Elevated	11.8 (14)			16.5 (28)		15.5 (38)		
High	15.1 (18)			25.2 (30)		14.7 (36)		
Very High	30.3 (36)			41.20 (49)		27.8 (68)		
Statistic	Chi^2^ = 2.6868 *p* = 0.846 C = 0.07							
**Pregnancy in the current epidemiological situation contributes to increased feelings of anxiety**	**I**			**II**		**III**		
	**% (n)**			**% (n)**		**%(n)**		
Yes	57.2 (68)			56.5 (96)		60 (147)		
No Opinion	11.8 (14)			13.5 (23)		17.1 (42)		
No	31.1 (37)			30.0 (51)		22.9 (56)		
Statistic	Chi^2^ = 43.5963 *p* = 0.008 C = 0.2747							
**Completion of the pregnancy in** **accord with prior ideas/plans would lessen feelings of anxiety.**	**Opinion**							
	**Yes**			**No Opinion**		**No**		
	**Average Rank**	**Me**		**Average Rank**	**Me**	**Average Rank**		**Me**
Childbirth Anxiety	351.05	15.00		246.56	14.00	265.72		18.00
Statistic	*p* = 0.004							

(Me)—median, (M)—mean, (SD)—standard deviation.

**Table 3:** Opinions of the respondents on specific sources of support and methods of treatment, by pregnancy trimester.

Source of Support/Factors	Pregnancy Trimester	Yes	No Opinion/ Not Applicable	No	Statistic
		% (n)	% (n)	% (n)	
In the current epidemiological situation, the support of the doctor in charge of the pregnancy was appropriate	I	76.5 (91)	16.8 (20)	6.7 (8)	Chi^2^ = 10.2185
	II	79.4 (135)	7.6 (13)	12.9 (22)	*p* = 0.036
	III	75.5 (185)	9.8 (24)	14.7 (36)	C = 0.1370
In the current epidemiological situation, the support of the midwife providing prenatal education was appropriate	I	42.0 (50)	52.9 (63)	5.0 (6)	Chi^2^ = 28.9580
	II	31.2 (53)	52.9 (90)	15.8 (27)	*p* = 7.972
	III	42.4 (104)	34.7 (85)	22.8 (56)	C = 0.2268
The support of loved ones (partner, family, friends) is important	I	95.8 (114)	3.4 (4)	0.8 (1)	Chi^2^ = 11.0432
	II	100 (170)	-	-	*p* = 0.026
	III	99.2 (243)	0.8 (2)	-	C = 0.1423
I am receiving sufficient support from my loved ones	I	91.6 (109)	3.4 (4)	5.0 (6)	Chi^2^ = 1.5008
	II	90.6 (154)	2.9 (5)	6.5 (11)	*p* = 0.826
	III	89.4 (219)	2.4 (6)	8.2 (20)	C = 0.0529
My marital/partnership relations have an influence on the level of childbirth anxiety	I	86.5 (103)	7.6 (9)	5.9 (7)	Chi^2^ = 7.1569
	II	91.8 (156)	4.1 (7)	4.1 (7)	*p* = 0.127
	III	93.5 (229)	2.0 (5)	4.5 (11)	C = 0.1150
The presence of a companion during childbirth helps to lessen perinatal anxiety	I	77.3 (92)	13.4 (16)	9.2 (12)	Chi^2^ = 10.0701
	II	78.2 (133)	18.8 (32)	3.0 (5)	*p* = 0.089
	III	78.8 (193)	13.9 (34)	7.3 (18)	C = 0.1360
Birthing school/prenatal education prepares you for childbirth physically	I	61.4 (73)	25.2 (30)	13.4 (16)	Chi^2^ = 16.5734
	II	56.5 (96)	33.5 (57)	10.0 (17)	*p* = 0.002
	III	47.3 (116)	29.4 (72)	23.3 (57)	C = 0.1734
Birthing school/prenatal education prepares you for childbirth psychologically	I	71.4 (85)	21.0 (25)	7.5 (9)	Chi^2^ = 2.7928
	II	72.4 (123)	22.9 (39)	4.7 (8)	*p* = 0.593
	III	69.3 (170)	21.6 (53)	8.9 (22)	C= 0.0721
The subject of childbirth anxiety was brought up during meetings with the midwife/in birthing school	I	33.6 (40)	55.5 (66)	10.9 (13)	Chi^2^ = 11.9339
	II	34.7 (59)	58.2 (99)	7.1 (12)	*p* = 0.017
	III	45.7 (112)	42.9 (105)	11.5 (28)	C = 0.1478
Birthing school/prenatal education helps for coping with perinatal anxiety	I	55.5 (66)	32.8 (39)	11.8 (14)	Chi^2^ = 0.7100
	II	57.0 (97)	34.1 (58)	8.9 (15)	*p* = 0.950
	III	57.9 (140)	33.1 (81)	9.8 (24)	C = 0.0364
The subject of SARS-CoV-2 (coronavirus) was brought up during meetings/teleconferences with the family midwife or in classes at the birthing school	I	16.8 (20)	77.3 (92)	9.36 (7)	Chi^2^ = 31.9594
	II	17.6 (30)	75.9 (129)	13.37 (11)	*p* = 0.001
	III	36.7 (90)	53.5 (131)	19.27 (24)	C = 0.2376
The current epidemiological situation was discussed during meetings/video conferences with the family midwife or in classes at the birthing school	I	17.6 (21)	78.2 (98)	1.7 (5)	Ch^2^ = 33.8456
	II	18.2 (31)	75.3 (128)	1.8 (11)	*p* < 0.001
	III	38.8 (95)	53.1 (130)	4.1 (20)	C = 0.2441
Perinatal care has an influence on feelings of childbirth anxiety	I	73.1 (87)	21.0 (25)	5.8 (7)	Chi^2^ = 10.4824
	II	78.8 (134)	18.8 (32)	2.4 (4)	*p* = 0.033
	III	79.2 (194)	12.7 (31)	8.1 (20)	C = 0.1387
Knowing about the standards of perinatal care helps in coping with anxiety	I	58.8 (70)	31.1 (37)	10.1 (12)	Chi^2^ = 18.2418
	II	75.2 (128)	15.9 (27)	8.9 (15)	*p* = 0.001
	III	79.2 (194)	13.1 (32)	7.7 (19)	C = 0.1817
Concluding the pregnancy by means of Cesarean section would lessen anxiety	I	31.1 (37)	14.3 (17)	54.6 (65)	Chi^2^ = 5.0353
	II	26.4 (45)	22.4 (38)	51.2 (87)	*p* = 0.283
	III	23.3 (57)	19.2 (47)	57.5 (141)	C = 0.0966

**Table 4:** Differences in respondents’ opinions on social support and factors influencing childbirth anxiety.

	**Sources of Support**					
**Psychological Condition**	**Loved Ones**		**Attending Physician**		**Family Midwife Providing Prenatal Education**	
	**Average Rank**	**Me**	**Average Rank**	**Me**	**Average Rank**	**Me**
Bad	208.04	4.00	228.13	4.00	226.22	3.00
Good	283.64	5.00	278.19	4.00	278.70	3.00
Statistic	Z = −5.16	*p* = 0.001	Z = −3.27	*p* = 0.001	Z = −3.40	*p* = 0.001
**Physical Condition**	**Loved Ones**		**Attending Physician**		**Family midwife providing prenatal education**	
	**Average Rank**	**Me**	**Average Rank**	**Me**	**Average Rank**	**Me**
Bad	261.43	4.00	249.70	4.00	241.72	3.00
Good	272.42	5.00	281.92	4.00	288.38	3.00
Statistic	Z = −0.91	*p* = 0.363	Z = −2.56	*p* = 0.011	Z = −3.67	*p* < 0.001
	**Factors Affecting Childbirth Anxiety**					
**Manner of pregnancy conclusion**	**Perinatal care and** **Birthing school**		**Pregnancy in the current epidemiological situation contributes to increased feelings of anxiety**		**Concluding the pregnancy by meansof Cesarean section would lessen anxiety**	
	M	SD	M	SD	M	SD
Not Applicable	22.69	3.50	3.65	1.20	2.48	1.31
Delivery without Complications	22.15	4.24	3.17	1.37	2.01	1.21
Delivery with Complications	22.25	3.96	3.50	1.33	3.12	1.48
Statistic	F = 1.09	*p* = 0.336	F = 5.34	*p* = 0.005	F = 19.95	*p* < 0.001
**Participation in** **Childbirth Classes**	**Perinatal care and** **Birthing school**		**Pregnancy in the current epidemiological situation contributes to increased feelings of anxiety**		**Concluding the pregnancy by means** **of Cesarean section** **would lessen anxiety**	
	**M**	**SD**	**M**	**SD**	**M**	**SD**
Yes	24.13	3.76	3.58	1.25	2.46	1.39
No	21.60	3.37	3.49	1.29	2.58	1.38
Statistic	T = 7.98	*p* < 0.001	T = 0.80	*p* = 0.426	T = −0.95	*p* = 0.341

(Me)—median, (M)—mean, (SD)—standard deviation.

**Table 5:** Differences in assessment of childbirth anxiety and factors influencing it.

**Psychological Condition**	**Childbirth Anxiety**			
	**Anxiety as a State**		**Anxiety as a Trait**	
	**M**	**SD**	**M**	**SD**
Bad	48.75	7.38	49.23	6.46
Good	39.75	8.03	40.00	8.20
Statistic	T = 10.80	*p* < 0.001	T = 12.72	*p* < 0.001
**Physical Condition**	**Anxiety as a State**		**Anxiety as a Trait**	
	**M**	**SD**	**M**	**SD**
Bad	44.13	8.57	44.95	8.30
Good	39.68	8.32	39.55	8.31
Statistic	T = 6.05	*p* < 0.001	T = 7.48	*p* < 0.001
**The course of the previous birth**	**Anxiety as a State**		**ANXIETY AS A Trait**	
	**M**	**SD**	**M**	**SD**
Not Applicable	41.57	8.42	42.12	8.67
Delivery without Complications	41.79	8.62	41.76	8.78
Delivery with Complications	41.84	9.52	41.77	8.86
Statistic	F = 0.05	*p* = 0.948	F = 0.10	*p* = 0.901
**Participation in** **Childbirth Classes**	**Anxiety as a State**		**Anxiety as a Trait**	
	**M**	**SD**	**M**	**SD**
Yes	41.21	9.04	40.80	8.77
No	41.94	8.52	42.64	8.63
Statistic	T = −0.92	*p* = 0.356	T = −2.35	*p* = 0.019

(M)—mean, (SD)—standard deviation.

**Table 6:** Analysis of the correlation between childbirth anxiety and the assessment of support from medical personnel and maternal experience.

Factors		Anxiety as a State	Anxiety as a Trait
Support during the current epidemiological situation from the OB-GYN treating the pregnancy	r	−0.15	−0.17
	*p*	<0.001	<0.001
Support during the current epidemiological situation from a family midwife providing prenatal education	r	−0.13	−0.18
	*p*	0.002	<0.001
Maternal experience	rho	0.01	−0.02
	*p*	0.882	0.628
Number of pregnancies	rho	0.02	−0.01
	*p*	0.653	0.860

r—Pearson’s correlation coefficient, rho—Spearman’s rho.

## Data Availability

Data are available upon reasonable request.

## Supplementary Materials

The following supporting information can be downloaded at: https://www.mdpi.com/article/10.3390/ijerph19052603/s1, Supplementary File: The Childbirth Anxiety Questionnaire.
