# Supplementary material for: Perinatal Anxiety among Women during the COVID-19 Pandemic—A Cross-Sectional Study
Source: Int J Environ Res Public Health. 2022 Feb 24;19(5):2603. doi: 10.3390/ijerph19052603 (PMC8909536; doi:10.3390/ijerph19052603)
Supplement: Supplementary file 1 [file ijerph-19-02603-s001.zip › ijerph-1547293-supplementary.pdf]

The Childbirth Anxiety Questionnaire (CAQ):

Emotions play an important role in childbirth. As you are expecting a baby, I am interested in how you feel about the labour today.

Please choose and mark the answer which best describes how you feel.

	Factors	*definitely yes*	*probably yes*	*probably not*	*definitely not*
1	I am afraid that my labour can be long				
2	I know that during the labour I will be able to completely control the situation.				
3	I am worried that my baby may be born with a birth defect				
4	I feel that some unforeseen complications may occur during my labor				
5	I am convinced that during the labour I will be calm and composed				
6	I am afraid that my labour will be painful				
7	I am convinced that I will recover quickly after giving birth				
8	I am worried that my baby may be harmed during childbirth				
9	Waiting for the birth is a very happy time for me				

State-Trait Anxiety Inventory for Adults Scoring Key (Form Y-1, Y-2)

Developed by Charles D. Spielberger in collaboration with R.L. Gorsuch, R. Lushene, P.R. Vagg, and G.A. Jacobs

To use this stencil, fold this sheet in half and line up with the appropriate test side, either Form Y-1 or Form Y-2. Simply total the scoring **weights** shown on the stencil for each response category. For example, for question # 1, if the respondent marked 3, then the **weight** would be **2**. Refer to the manual for appropriate normative data.

Form Y-1	NOT AT ALL	SOMEWHAT	MODERATELY SO	VERY MUCH SO
1.	4	3	2	1
2.	4	3	2	1
3.	1	2	3	4
4.	1	2	3	4
5.	4	3	2	1
6.	1	2	3	4
7.	1	2	3	4
8.	4	3	2	1
9.	1	2	3	4
10.	4	3	2	1
11.	4	3	2	1
12.	1	2	3	4
13.	1	2	3	4
14.	1	2	3	4
15.	4	3	2	1
16.	4	3	2	1
17.	1	2	3	4
18.	1	2	3	4
19.	4	3	2	1
20.	4	3	2	1

Form Y-2	ALMOST NEVER	SOMETIMES	OFTEN	ALMOST ALWAYS
21.	4	3	2	1
22.	1	2	3	4
23.	4	3	2	1
24.	1	2	3	4
25.	1	2	3	4
26.	4	3	2	1
27.	4	3	2	1
28.	1	2	3	4
29.	1	2	3	4
30.	4	3	2	1
31.	1	2	3	4
32.	1	2	3	4
33.	4	3	2	1
34.	4	3	2	1
35.	1	2	3	4
36.	4	3	2	1
37.	1	2	3	4
38.	1	2	3	4
39.	4	3	2	1
40.	1	2	3	4

© Copyright 1968,1977 by Charles D. Spielberger. All rights reserved.
Published by Mind Garden, Inc., 1690 Woodside Rd, Suite 202, Redwood City, CA 94061

Copyright 1968, 1977 by Charles D. Speilberger. All rights reserved.

SELF-EVALUATION QUESTIONNAIRE

STAI Form Y-2

Name____ Date__________

DIRECTIONS

A number of statements which people have used to describe themselves are given below. Read each statement and then circle the appropriate number to the right of the statement to indicate how you *generally* feel. There are no right or wrong answers. Do not spend too much time on any one statement but give the answer which seems to describe how you generally feel.

	ALMOST NEVER	SOMETIMES	OFTEN	ALMOST ALWAYS
21. I feel pleasant	1	2	3	4
22. I feel nervous and restless	1	2	3	4
23. I feel satisfied with myself	1	2	3	4
24. I wish I could be as happy as others seem to be	1	2	3	4
25. I feel like a failure	1	2	3	4
26. I feel rested	1	2	3	4
27. I am "calm, cool, and collected"	1	2	3	4
28. I feel that difficulties are piling up so that I cannot overcome them	1	2	3	4
29. I worry too much over something that really doesn't matter	1	2	3	4
30. I am happy	1	2	3	4
31. I have disturbing thoughts	1	2	3	4
32. I lack self-confidence	1	2	3	4
33. I feel secure	1	2	3	4
34. I make decisions easily	1	2	3	4
35. I feel inadequate	1	2	3	4
36. I am content	1	2	3	4
37. Some unimportant thought runs through my mind and bothers me	1	2	3	4
38. I take disappointments so keenly that I can't put them out of my mind	1	2	3	4
39. I am a steady person	1	2	3	4
40. I get in a state of tension or turmoil as I think over my recent concerns and interests	1	2	3	4

Copyright 1968, 1977 by Charles D. Speilberger. All rights reserved.

© Copyright 1968,1977 by Charles D. Spielberger. All rights reserved.
Published by Mind Garden, Inc., 1690 Woodside Rd, Suite 202, Redwood City, CA 94061
